# The Aryl Hydrocarbon Receptor in Neurotoxicity: An Intermediator Between Dioxins and Neurons in the Brain

**DOI:** 10.3390/toxics13070596

**Published:** 2025-07-16

**Authors:** Eiki Kimura

**Affiliations:** Department of Environmental Health, University of Fukui School of Medical Sciences, 23-3 Matsuoka-shimoaizuki, Eiheiji, Fukui 910-1193, Japan; kimuraei@u-fukui.ac.jp; Tel.: +81-776-61-8337

**Keywords:** aryl hydrocarbon receptor, brain, dioxin, neuron, neurotoxicity

## Abstract

Industrial development has increased environmental dioxin concentrations, sparking concern about human health impacts. Examining dioxin neurotoxicity has highlighted associations with cognitive impairment and behavioral abnormality. Dioxins are ligands of the aryl hydrocarbon receptor (AHR), a ligand-activated transcription factor; it is speculated that dioxin-induced AHR activation is pivotal for toxic effects. Accurate AHR-expressing cell identification is therefore indispensable for understanding the molecular and cellular mechanisms of dioxin toxicity. Herein, current knowledge regarding AHR expression in the mammalian brain is summarized, and dioxin neurotoxicity mechanisms are discussed. Histological studies show AHR-expressing neurons in multiple brain regions, including the hippocampus and cerebral cortex. Dopaminergic and noradrenergic neurons exhibit AHR expression, suggesting possible roles in the monoaminergic system. AHR overactivation evokes dendritic arborization atrophy, whereas its deficiency increases complexity, implying that AHR-mediated signaling is crucial for neuronal growth and maturation. AHR is also involved in neurogenesis and neuronal precursor migration. Collectively, these findings support the notion that dioxin-induced AHR overactivation in individual neurons disrupts neural circuit structure, ultimately leading to impaired brain function. However, as AHR downstream signaling is intertwined with various molecules and pathways, the precise mechanisms remain unclear. Further studies on the expression, signaling, and roles of AHR are needed to clarify dioxin neurotoxicity.

## 1. Introduction

**Figure 1:**
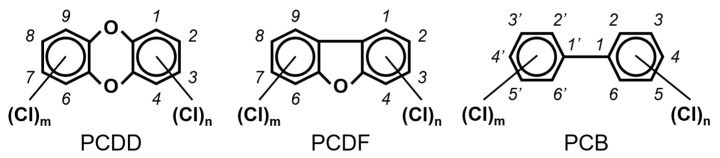
The chemical structures of polychlorinated dibenzo-*p*-dioxin (PCDD), polychlorinated dibenzofuran (PCDF), and polychlorinated biphenyl (PCB).

Chlorinated dioxins include two series of organohalogenated substances, polychlorinated dibenzo-*p*-dioxins and dibenzofurans (PCDD/Fs), which together contain 210 congeners (75 PCDDs and 135 PCDFs). Chemically, the compounds comprise two benzene rings connected by oxygen atoms, differing in the number and position of their chlorine atoms (Figure 1). Because dioxins can be formed by natural processes, such as forest fires and volcanic eruptions [1,2], they were present on Earth long before industrialization. For instance, dioxins were identified in historical soil, herbage, and sediment samples collected from England and Switzerland in the 1840s [3,4]. They were also identified in soil samples dating back to 70–40 B.C., sourced from Roman brickwork along the Lower Rhine in Germany [5]. With the development of the chlorine chemical industry in the 20th century, dioxins were produced as unwanted byproducts of industrial and thermal processes and released into the environment, substantially increasing their concentrations in our surroundings [1,3,4,6]. Polychlorinated biphenyls (PCBs), comprising 209 congeners, are also organohalogenated substances that are deeply related to dioxins (Figure 1), because some coplanar congeners have similar toxicological characteristics (i.e., dioxin-like PCBs (dl-PCBs)). In contrast to dioxins, PCBs began to be produced commercially in 1929 and had a wide range of applications, such as electronic appliances and heat transfer systems. Although their production is currently banned, they may be produced unintentionally during thermal reactions in industrial processes [2,7].

Dioxins and PCBs, which are both lipophilic and hydrophobic, are chemically stable and resistant to metabolic processes, resulting in environmental persistence. Humans have a relatively high trophic level; as these compounds tend to bioaccumulate, human tissues may contain relatively high amounts of them. Therefore, humans are at risk of not only occupational and accidental exposure but also environmental (background) exposure. A plethora of epidemiological, clinical, and experimental studies has revealed a variety of toxic effects in humans and laboratory animals, including cancer, reproductive disorders, immune deficiency, endocrine disruption, and cognitive impairment [2]. Importantly, molecular studies have determined that most, if not all, of the toxic effects of exposure to dioxins and dl-PCBs are evoked through the aryl hydrocarbon receptor (AHR), a ligand-activated transcription factor. Upon binding to a ligand, AHR translocates from the cytoplasm into the nucleus, where it acts as a transcription factor, inducing target gene expression [8]. Because some dioxins and dl-PCBs function as high-affinity ligands, AHR is a pivotal molecule in their toxicity.

A meeting of the World Health Organization was held in 1997 to assess and propose toxic equivalency factor (TEF) values for a group of 29 dioxin and dl-PCB congeners based on their chemical structure, binding affinity to and reactivity with AHR, environmental persistence, and bioaccumulation in the food chain [9]. Specifically, seventeen dioxins (seven PCDDs and ten PCDFs) displaying 2,3,7,8-substituted compounds with tetra- to octa-chlorines and twelve dl-PCBs (non- and mono-*ortho*-substituted PCBs) were assigned TEF values indicating their toxic strength relative to 2,3,7,8-tetrachlorodibenzo-*p*-dioxin (TeCDD, TEF = 1), the most toxic congener in the group. These TEF values have since been reevaluated and revised twice, in 2005 and 2022 [10,11] (Table 1), and the risk posed by dioxins is still a subject of active global debate.

Accumulating evidence suggests that a variety of chemicals, including organohalogenated substances, could potentially disrupt the development and function of the central nervous system (CNS) in children [12,13,14,15]. Because dioxin exposure reportedly evokes impaired brain function and abnormal behavior in humans and laboratory animals, dioxins are representative chemicals that contribute to the disruption of developmental processes in the CNS, such as neuronal growth and neural circuit formation. Thus, the goals of this review are to (1) outline epidemiological studies showing dioxin neurotoxicity associated with human brain function, (2) summarize experimental studies using laboratory animals that support dioxin neurotoxicity in humans, and (3) review AHR expression and signaling with an emphasis on neurons in the mammalian brain.

## 2. Epidemiological Studies on Dioxin Neurotoxicity

In previous decades, epidemiological studies have revealed the toxic effects of dioxins on neurodevelopment in humans. First, food poisoning caused by rice bran oil contaminated with the dioxins generated from heat-degraded PCBs was identified. Known as “oil disease,” it was reported in Yusho, Japan, in 1968 and Yu-cheng, Taiwan, in 1979. Patients suffering from oil disease exhibited various symptoms, such as hyperpigmentation and ophthalmological manifestations [16,17,18,19]. Because some neurological symptoms, including mild intellectual disability, were reported in children born to mothers consuming contaminated oil in Yusho [20], it is plausible that dioxin exposure could have disrupted the development of the CNS during the fetal and infant stages. Neurotoxic effects were also reported in the children of mothers exposed to dioxins and PCBs in Yu-cheng. Specifically, worse scores in multiple landmarks associated with mental and motor development were revealed in exposed children aged 1–82 months compared to unexposed children [21]. Later, cognitive impairment and activity disorders were found in children up to 12 years of age [22,23,24,25,26,27]. While dioxin congeners, especially 2,3,4,7,8-pentachlorodibenzofuran, are likely involved in toxic effects in patients [16,28], it is interesting to note that perinatal exposure to PCBs is relevant to cognitive and intellectual impairments in children [29,30,31]. Therefore, PCBs could partly contribute to neurotoxicity, suggesting that combined exposure is an underlying mechanism of toxicities in oil disease.

During the Vietnam War (1961–1971), the herbicide Agent Orange, which contains dioxins, was sprayed in Southern Vietnam by the United States Air Force (USAF) [32,33]. Moreover, owing to herbicide spills, dioxins have contaminated the environment (i.e., soil and sediment) surrounding former USAF bases [34]. Thus, it is reasonable to speculate that dioxins could be transferred not only to aquatic organisms, such as fish, but also to agricultural products, resulting in human exposure through the food chain. Consistent with this speculation, dioxin levels in the breast milk of mothers residing in contaminated areas around former USAF bases were higher than those of mothers in unsprayed areas [35,36], implying that their infants were perinatally exposed to dioxins. Indeed, birth cohort studies have revealed altered body growth and adverse effects on neurodevelopment, such as cognitive, language, and motor functions, in infants and children [37,38,39]. Additionally, epidemiological evidence of children exhibiting symptoms of neurodevelopmental disorders (i.e., autistic traits, learning disabilities, and hyperactivity) in contaminated areas continues to accumulate [40,41,42]. Notably, among the dioxin congeners, TeCDD is strongly associated with these symptoms. Magnetic resonance imaging scans of adult men who were estimated to have been perinatally exposed to dioxins displayed increases in gray matter volume and total brain volume, while their white matter volume was decreased [43,44,45]. Intriguingly, these studies also demonstrated relationships between brain volume and blood dioxin levels, a marker of exposure in adulthood. This suggests the possibility of dioxin exposure impacting brain structure and function even in adulthood. In line with this idea, veterans exposed to Agent Orange reportedly exhibited brain atrophy and a higher prevalence of dementia than unexposed veterans in Korea and the USA [46,47].

Epidemiological studies in European countries and the USA have revealed the effects of perinatal exposure to dioxins on neurodevelopment in children. A chemical factory explosion in Seveso, Italy, occurred in 1976, exposing residents to dioxins [48]. In the beginning, while cancer prevalence and reproductive impairment increased, no overt neurotoxicity to physical function or working memory were found [49]. Later, another study focusing on second-generation health revealed that exposure adversely affected neuropsychological functions in boys, albeit with limited evidence [50]. Notably, several studies showing dioxin neurotoxicity at a background level have uncovered that higher dioxin concentrations in mothers (i.e., in blood and breast milk) are correlated with worse scores associated with learning, attention, language skills, and motor development in the Netherlands, Denmark, Norway, Germany, and the USA [51,52,53,54,55,56,57]. Two cohort studies in Japan suggested possible effects of environmental dioxins on health conditions, including neurodevelopmental conditions [58]. Dioxin concentrations in maternal samples were inversely correlated with the mental and psychomotor development of their children at 6 months [59,60], whereas little effect was found at 18 and 42 months [61,62].

These epidemiological studies have demonstrated adverse effects of dioxins on brain development and function in humans, suggesting the CNS as a target tissue. Generally, in the case of human studies, it is extremely difficult to completely exclude interactions between other factors, such as co-exposure to multiple chemicals and the lifestyles of individuals; therefore, experimental studies using laboratory animals are helpful to confirm the toxicity of specific chemicals. In the next section, I summarize the current understanding of behavioral abnormalities in rodents exposed to dioxins to assess the neurotoxicity.

## 3. Experimental Animal Studies on Dioxin Neurotoxicity

In the field of neuroscience, various behavioral tests have been developed to assess multiple brain functions, such as learning and memory, emotion, and motor activity, in laboratory animals, especially in rats and mice [63,64]. Toxicological researchers actively utilized these tests to evaluate the neurotoxicity of environmental pollutants and the safety of pharmaceutical chemicals [65]. In parallel, histopathological examination focusing on neuronal morphology offers valuable insights into behavior, because neuronal growth and neural circuit structure are linked to the development and function of the CNS. For example, neural circuits in the hippocampal CA1, CA3, and dentate gyrus (DG) subregions are known to be crucial for learning and memory in both humans and rodents [66]. Moreover, aberrant neuromorphology has been found in the brains of human patients and animal models with neurodevelopmental disorders and age-related diseases [67,68,69,70]. These results indicate the deep relationship between brain functions and neural circuit structures, supporting the importance of behavioral assessment with simultaneous histopathological examination to more accurately evaluate chemical neurotoxicity.

Rodent offspring born to dams administered dioxins have been frequently used for behavioral tests, revealing the impairment of multiple brain functions later in life after in utero and lactational exposure (Table 2). Specifically, perinatal exposure to TeCDD induced learning and memory impairment in not only rodents [71,72,73,74,75,76,77,78] but also in non-human primates [79]. Furthermore, rat and mouse offspring exposed to TeCDD in utero and via lactation exhibited impairments of contextual fear memory, behavioral flexibility, operant behavior, and motor behavior [80,81,82,83,84,85], as well as hyperactivity [86], in adulthood. Atypical behaviors have been reported in not only adults but also infants. My colleagues and I revealed the suppression of ultrasonic vocalizations (USVs) emitted by the infant mice of dams treated with TeCDD [87]. Because atypical infant USV emissions have been detected in several mouse models with neurodevelopmental disorders [88,89,90], our study suggests that infant USVs are toxicologically valuable as a behavioral endpoint at an early life stage. In line with epidemiological studies, these resultant abnormalities in laboratory animals support the notion that the exposure of mothers to dioxins adversely affects the development and function of the CNS in their offspring.

Such behavioral abnormalities could potentially be caused by dioxins disrupting the neural circuit structure. Indeed, perinatal exposure to TeCDD altered the expression pattern of key genes regulating neurite elongation and synapse formation in the developing brains of mouse offspring [91,92]. Histopathological examination demonstrated the atrophy of the cerebral cortex in mice perinatally exposed to TeCDD, along with a decrease in non-GABAergic neurons [93]. Aberrant morphology was found in the hippocampal and amygdala neurons of TeCDD-exposed mouse offspring [71,94]. This is consistent with an in vitro study demonstrating TeCDD-dependent changes in neurite elongation in primary cultured cortical neurons [95]. Additionally, perinatal exposure to TeCDD inhibited neurogenesis in the cerebellar granule neurons of developing mice [96]. These findings are in accordance with the idea that in utero and lactational exposure to dioxins may disrupt brain development, including neuronal growth, in the fetal and infant stages, ultimately leading to impaired brain function later in life.

Dioxin exposure, even in adulthood, can elicit behavioral abnormalities. TeCDD exposure reduced neurogenesis in the hippocampal DG of adult mice, exhibiting the impairment of contextual fear memory [97]. This implies that the hippocampus is vulnerable to dioxins and, importantly, is consistent with the epidemiological study reporting the increased prevalence of dementia in veterans described above [47]. In another study, depression-like behavior was observed in mice exposed to TeCDD [98]. These studies suggest the potential risk of dioxins to brain function and neural circuit maintenance throughout life.

Behavioral tests using laboratory animals are valuable tools to assess brain function and evaluate dioxin neurotoxicity; however, there are large differences in behavioral styles between humans and other species. When attempting to compensate for this gap and obtain compelling evidence, a molecular biology perspective is helpful. In this context, AHR, a protein that functions as a dioxin receptor, is key to elucidating the toxic mechanisms of dioxins. Therefore, in the following section, I briefly outline the profile of AHR.

**Table 2:** Behavioral abnormalities of laboratory animals exposed to 2,3,7,8-substituted dioxins.

Dioxins	Chemical Structure	TEFs	Behaviors (Brain Functions)	Species	References
TeCDD	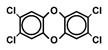	1	Learning and memory	Monkey	Schantz and Bowman, 1989 [79]
				Rat	Schantz et al., 1996 [75]
					Seo et al., 1999 [77]
					Seo et al., 2000 [76]
					Hojo et al., 2008 [73]
					Kakeyama et al., 2014 [74]
					Zhang et al., 2018 [78]
					Hattori et al., 2021 [72]
				Mouse	Gileadi et al., 2021 [71]
			Contextual fear memory	Rat	Mitsui et al., 2006 [83]
				Mouse	Haijima et al., 2010 [81]
					Latchney et al., 2013 [97]
			Operant behavior	Rat	Markowski et al., 2001 [82]
			Motor behavior	Rat	Nishijo et al., 2007 [85]
			Socioemotional behavior	Rat	Nguyen et al., 2013 [84]
			Behavioral flexibility	Mouse	Endo et al., 2012 [80]
			Infant USV	Mouse	Kimura and Tohyama, 2018 [87]
			Hyperactivity	Mouse	Sha et al., 2021 [86]
			Depression-like behavior	Mouse	Debler et al., 2024 [98]
TeBDD	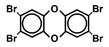	1 ^a^	Learning and memory	Rat	Kakeyama et al., 2014 [74]
			Contextual fear memory	Mouse	Haijima et al., 2010 [81]
TeBDF	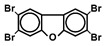	0.1 ^a^	Exploratory behavior	Mouse	Kimura et al., 2020 [99]
			Infant USV	Mouse	Kimura et al., 2020 [99]
					Kimura et al., 2022 [100]
TrBCDF	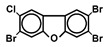	0.1 ^a^	Infant USV	Mouse	Kimura et al., 2023 [101]

^a^ Interim values based on Van den Berg et al., 2013 [102]. TEF, toxic equivalency factor; TeBDD, 2,3,7,8-tetrabromodibenzo-*p*-dioxin; TeBDF, 2,3,7,8-tetrabromodibenzofuran; TeCDD, 2,3,7,8-tetrachlorodibenzo-*p*-dioxin; TrBCDF, 2,3,7-tribromo-8-chlorodibenzofuran; USV, ultrasonic vocalization.

## 4. Molecular Characteristics of AHR

**Figure 2:**
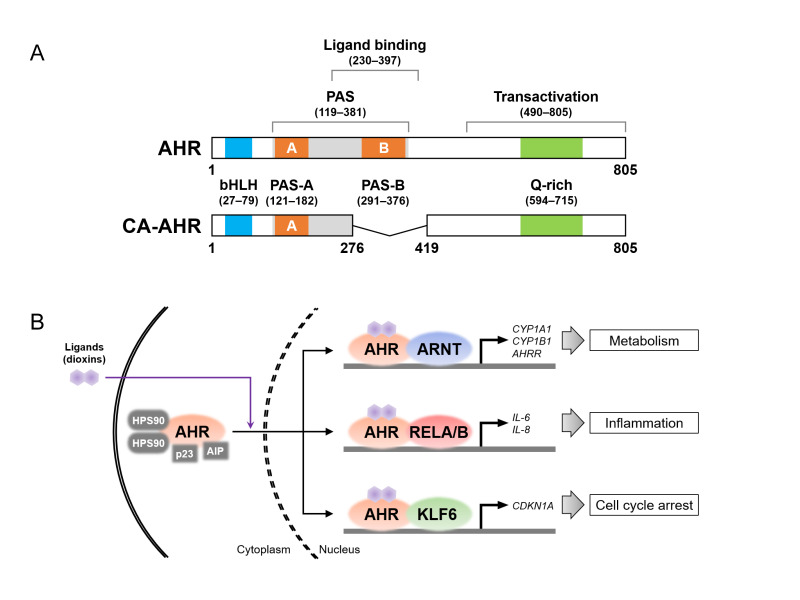
Aryl hydrocarbon receptor (AHR) protein structure and its binding partners. (**A**) Protein structures of the full-length AHR in the C57BL/6 mouse strain (**top**) and constitutively active (CA) AHR (**bottom**). AHR consists of the functional domains, i.e., Per-Arnt-Sim (PAS, shown in gray), ligand-binding, and transactivation domains. The basic helix–loop–helix (bHLH), PAS-A and -B, and glutamine (Q)-rich motifs are shown in blue, orange, and green, respectively. The CA-AHR shows the deletion of amino acid residues 277–418, containing the ligand-binding domain and the PAS-B motif. The number of amino acid residues in each domain and motif were sourced from the references [94,125,126,143,144]. (**B**) Schematic image of ligand-induced nuclear translocation of AHR and AHR complexes with partner proteins (i.e., AHR nuclear translocator (ARNT), RELA/B, and Krüppel-like factor 6 (KLF6)) for the transcriptional induction of their target genes. AHRR, AHR repressor; AIP, AHR-interacting protein; CDKN1A, cyclin-dependent kinase inhibitor 1A; HSP90, heat shock protein 90.

AHR, a member of the basic helix–loop–helix (bHLH)/Per-Arnt-Sim (PAS) family, consists of multiple domains, including the PAS and transactivation domains (Figure 2A), which were cloned in humans and mice in the 1990s [103,104]. As mentioned in the introduction, AHR is localized in the cytoplasm, forming a complex with heat shock protein 90, AHR-interacting protein (also known as XAP), and p23 in the absence of its ligand [105]. Interestingly, varieties of AHR ligands have been identified not only in environmental pollutants, such as dioxins, but also in dietary and microbial metabolites [106,107]. The liganded AHR translocates to the nucleus, where it forms another complex with nuclear proteins and acts as a transcription factor, upregulating the expression of various target genes (Figure 2B). To date, several nuclear proteins have been identified as AHR-binding partners, regulating cellular activities and physiological functions through the induction of specific target genes. First, the AHR nuclear translocator (ARNT), belonging to the bHLH-PAS family, is the most well-known partner protein [108]. The AHR–ARNT heterodimer induces the expression of the cytochrome P450 (CYP) family genes, which encode enzymes that metabolize xenobiotic chemicals [109]. The heterodimer binds to xenobiotic response element (XRE) sequences in the genomic DNA, upregulating the expression of *CYPs*, such as *CYP1A1* and *1B1*, and *AHR repressor* (*AHRR*) genes. AHRR belongs also to the bHLH-PAS family and competes with AHR to bind to ARNT; thus, AHR-dependent transcriptional activity is inhibited by AHRR, likely providing negative feedback for AHR signaling pathways [110]. Second, the nuclear factor (NF)-κB subunits (i.e., RELA and RELB), which control the inflammatory response, are also known partner proteins of AHR [111,112]. Functionally, the expression of interleukin-6 (IL-6) is upregulated by the activation of both AHR and RELA [113], while the AHR–RELB complex induces the expression of IL-8 in TeCDD-exposed cells [114]. These results suggest that some inflammatory responses are intertwined with the crosstalk between AHR- and NF-κB-signaling regulators. Third, AHR binds to Krüppel-like factor 6 (KLF6) for transcriptional activity, inducing the expression of cyclin-dependent kinase inhibitor 1A (CDKN1A, also known as p21^CIP1^) and plasminogen activator inhibitor-1 [115,116]. CDKN1A plays an important role in cell cycle arrest via hypophosphorylation in retinoblastoma (RB) [117], suggesting the possibility that AHR is associated with cellular proliferation through preferential interactions with KLF6. In line with this idea, exposure to xenobiotic AHR ligands induces cell cycle arrest and increases the expression of CDKNs, including CDKN1A [93,118,119,120]. Furthermore, AHR forms complexes with multiple proteins, such as the RB, Maf, and estrogen receptor proteins [121,122,123], which is consistent with the fact that numerous genes are regulated in an AHR-dependent manner [124]. The reader should refer to specialized reviews for further details regarding the signaling pathways linked to AHR, ligands, and partner proteins [125,126,127,128,129,130,131].

Given these characteristics of AHR, even in situations in which AHR needs to be stably localized in the cytoplasm, dioxins may induce forced translocation into the nucleus of exposed cells, resulting in excessive transcriptional activation. The subsequent overexpression of AHR target genes could potentially perturb intracellular signaling pathways, leading to the disruption of cellular growth, tissue structure, and organ function. Specifically, TeCDD exposure evoked tissue lesions and teratogenicity, such as cleft palates and hydronephrosis, in wild type (*Ahr^+/+^*) mice but not in *Ahr^−/−^* mice [132,133], indicating the pivotal role of AHR and its downstream signaling in dioxin toxicity. While exhibiting resistance to TeCDD, albeit not to its embryonic lethality, *Ahr^−/−^* mice displayed abnormalities in developmental processes, physiological functions, aging, and lifespan [134,135,136,137]. Intriguingly, loss-of-function mutations in *AHR* genes are considered the genetic etiology of congenital nystagmus in both humans and mice [138,139,140], suggesting a possible role of AHR in nervous system development across species. Mice lacking AHR exhibited learning and memory impairment, neurogenesis reduction, and synaptic dysfunction, whereas no obvious gross anatomical abnormalities were reported in their brain tissues [97,141,142]. These biological and clinical studies suggest neuronal AHR expression throughout life (i.e., from the embryonic to aged stages) and highlight the importance of the spatiotemporal regulation of AHR signaling pathways in the nervous system. Therefore, the accurate identification of AHR-expressing neurons is indispensable to understanding the molecular mechanism of dioxin neurotoxicity. Next, I summarize the current insights provided by studies on AHR expression in the mammalian brain.

## 5. Neuronal AHR in the Mammalian Brain

AHR expression in the mammalian brain has been examined at both the transcript and protein levels. In humans, *AHR* transcripts have been detected in adult and fetal brains using reverse transcription PCR (RT-PCR), albeit at a lower level than that in other tissues [145]. RT-PCR also revealed *Ahr* transcripts in multiple brain regions in rats and mice, including the hippocampus, cerebral cortex, cerebellum, olfactory bulb, and pituitary gland [146,147]. Consistent with the transcript-level results, AHR proteins were found in these brain regions in mice by western blotting [148]. However, while these bulk approaches strongly indicate AHR expression in the brain, it is impossible to use them to identify which neurons harbor AHR in situ.

Histological experiments are useful to microscopically identify the AHR-expressing regions and neurons in a tissue sample. In situ hybridization (ISH) revealed *Ahr* transcript expression in various brain regions in adult rats, including the hippocampus and cerebral cortex [149], which is consistent with the RT-PCR and western blotting results [146,147,148]. More specifically, the ISH study demonstrated *Ahr* transcripts in the hippocampal pyramidal and granule cell layers, which make up most of the neurons in the CA1, CA2, CA3, and DG subregions [149]. These neuronal layers have more abundant *Ahr* transcripts than nearby areas (i.e., the stratum radiatum and oriens in the CA1, CA2 and CA3, and the hilus in the DG), suggesting that AHR is preferentially expressed in neurons. Similar to rats, mice also exhibited high *Ahr* transcripts in the CA1, CA3, and DG neuronal layers in both adult and developmental stages [147]. Additionally, in mice, *Ahr* transcripts were found in the neuroepithelium, which is the neuronal precursor of embryos [150,151]; thus, AHR may serve a function in neurogenesis. In support of this speculation, even in adults, *Ahr* transcripts were detected in neuronal precursors in the hippocampal DG subregion [97] and in the rostral migratory stream (RMS), where neuronal precursors migrate toward the olfactory bulb [147]. These findings suggest that AHR contributes to the growth of neuronal lineage cells from the developmental to adult stages. The detection of *Ahr* alongside a neuronal marker provides compelling evidence of its neuronal expression. In rats, ISH revealed *Ahr* transcripts in *Gad1/2* transcript-positive cells (i.e., GABAergic neurons) in the preoptic area [152]; however, how AHR contributes developmentally and functionally to the GABAergic system in the mammalian brain remains unclear.

Using immunohistochemistry (IHC) to detect proteins is a more useful approach than ISH, because it allows researchers to not only identify AHR-expressing neurons but also analyze the intracellular localization of AHR at the single-neuron level. Notably, several IHC studies have shown hippocampal neurons harboring AHR across mammalian species. Consistent with ISH studies, AHR proteins were detected in the CA1, CA3, and DG neuronal layers of rodents [153,154,155], and in the CA1 neuronal layer in human brains [156]. While, in mice, AHR was found in the CA1 neuronal layer, its immunohistochemical staining concentration was dramatically attenuated by *Ahr* knockdown [141]. Additionally, AHR was detected in neurons expressing NeuN, a general neuronal marker, in the cerebral cortex and island of Calleja major (ICjM) in mice [148,157]. Intriguingly, in some IHC studies, AHR appears to be abundantly expressed in monoaminergic neurons. For instance, AHR was found in dopaminergic neurons expressing tyrosine hydroxylase (TH), an enzyme which is crucial for dopamine synthesis, in the ventral tegmental area and substantia nigra compacta of rodents [158,159]. In another study, AHR was evident in neurons expressing TH and dopamine-β-hydroxylase in the locus coeruleus (LC), responsible for the noradrenergic system, in *Ahr^+/+^* mice but not in *Ahr^−/−^* mice [148]. Moreover, microscopy image analysis revealed that nuclear AHR was significantly increased in neurons of the ICjM and LC in mice orally exposed to TeCDD [148]. This provides evidence that dioxin intake activates AHR in brain neurons after intestinal absorption, blood circulation, and the crossing of the blood–brain barrier. Taken together, these histological findings indicate the existence of AHR in multiple types of brain neurons, implying its fundamental role in brain development and function in mammals.

In vitro studies using primary cultured neurons are helpful in supporting the notion of neuronal AHR expression. Immunocytochemistry (ICC) and western blotting have demonstrated AHR expression in cultured neurons from the mouse hippocampus [160,161] in accordance with histological observations in human and rodent brains. RT-PCR, western blotting, and ICC have identified *Ahr* transcripts and AHR proteins in cultured neurons from the cerebral cortex and cerebellum of rodents [162,163,164,165,166]. Cultured neurons from the hippocampus and cerebral cortex have been shown to exhibit the co-expression of AHR and MAP-2, a neuron-specific microtubule [161,163]. Additionally, the HT22 cell line, originating from mouse hippocampal neurons, harbors AHR [154,167]. These in vitro studies provide experimental evidence of the neuronal expression of AHR in the mammalian brain.

Table 3 summarizes AHR-expressing neurons in the mammalian brain, with AHR expression patterns organized according to neuronal subtypes, brain regions, and species. Collectively, these histological studies have revealed AHR-expressing neurons in various brain regions. This fits well with the idea that dioxins disrupt the molecular processes of cellular growth and maturation through excessive AHR activation in individual neurons, finally leading to brain functional impairment. Because neuromorphology and neural circuit structure are strongly associated with brain function as described above, I will briefly discuss the potential impacts of AHR activation on neuronal growth.

## 6. Impact of Excessive AHR Activation on Neuronal Growth in the Brain

As mentioned earlier, both in vivo and in vitro studies have revealed aberrant neurite morphology in individual neurons following TeCDD exposure, as well as upregulated AHR target gene expression [71,92,94,95]. Additionally, AHR-deficient neurons have displayed altered dendritic arborization complexity [141]. In line with these findings, the TeCDD-induced nuclear translocation of AHR has been observed microscopically in brain neurons and primary cultured neurons [148,166], suggesting that AHR signaling is relevant to neuronal growth, especially neurite elongation.

Constitutively active (CA)-AHR can be used to examine the effect of AHR signaling activation on cellular growth and maturation. AHR that lacks the ligand-binding domain and the PAS-B motif functions as CA-AHR (Figure 2A), allowing the induction of transcriptional activity even in the absence of a ligand [169]. The transgenic mouse line expressing CA-AHR has been found to exhibit not only tumorigenesis and organ hypertrophy [170,171] but also the disruption of immune cells and reproductive organs [172,173], which seems at least partly similar to the toxic effects displayed by dioxin-exposed rodents [174]. In addition, in vivo electroporation is an excellent technique for transfecting plasmid vectors carrying genes encoding specific proteins, such as fluorescence proteins for cell visualization, into the neuronal lineage cells of developing mouse brains [175,176,177]. This technique allows researchers to microscopically observe neurons labeled with fluorescence proteins in brain tissues and to quantitatively analyze their morphology and location at the single-neuron level.

**Figure 3:**
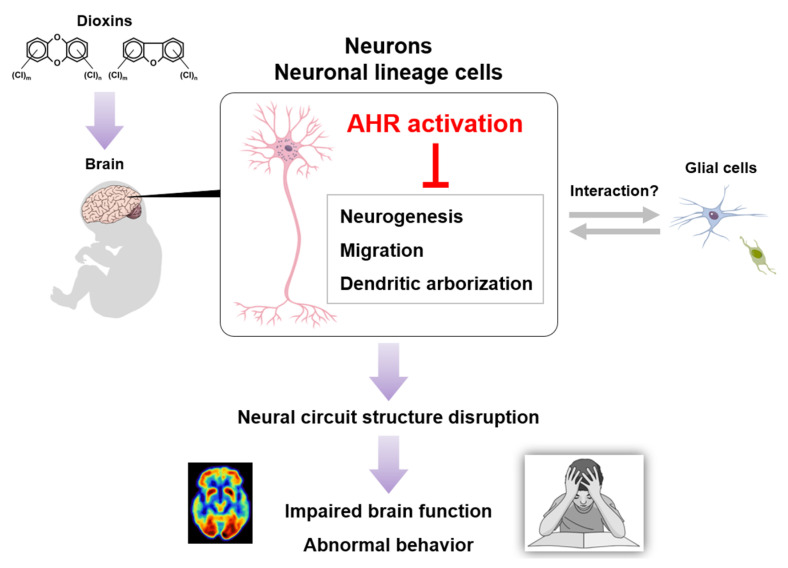
A possible model of dioxin neurotoxicity. Dioxin exposure induces excessive AHR activation in brain neurons, followed by neural circuit structure disruption, finally leading to impaired brain function and abnormal behavior.

My colleagues and I utilized these two genetic tools to examine mature neurons and neuronal precursors expressing CA-AHR together with fluorescence proteins and found aberrant dendritic morphology in CA-AHR-expressing pyramidal and granule neurons in the hippocampus, cerebral cortex, and olfactory bulb of infant mice [94,178,179]. It is plausible that these dendritic abnormalities are commonly induced by excessive AHR activation across brain regions and neuronal subtypes. Intriguingly, no overt dendritic changes were observed in neurons transfected with the vector carrying the gene encoding full-length AHR (i.e., AHR overexpression); thus, excessive AHR signaling activation (i.e., its constitutively active form) is key to the observed neuromorphological abnormalities. In addition to dendritic changes, CA-AHR morphologically disrupted the initial processes of neuronal precursors in the RMS [178]. In terms of the migrating precursor phenotype, the CA-AHR-expressing neurons could not reach their destinations and finally displayed the perturbation of their distributions within several brain regions [178,179,180]. Considered together, these findings suggest a potential mechanism in which excessive AHR signaling activation, caused by dioxins, disrupts neuronal growth, such as neurite elongation and cell migration (Figure 3).

## 7. Glial AHR in the Mammalian Brain

Neuron–glia interactions contribute to neural circuit maintenance and activity, as well as neurological disorders [181,182,183], suggesting that glial cells could be partly involved in AHR-related neurotoxicity. Consistent with this idea, several studies have reported that AHR is detectable in glial cells, in addition to neurons, of human and rodent brains (Table 4). Initially, AHR expression was found in human glioblastoma tissues [184]; therefore, AHR is likely associated with tumor progression via the activation of its signaling in glial cells. Indeed, AHR expression was clearly detected in glioblastoma regions in a biopsy, while its expression was low in non-tumor regions [184]. Supporting this observation, correlations between AHR expression and cancer severity grades were found in human glioma and meningioma [185,186,187]. However, AHR expression in astrocytes and microglia has been demonstrated by IHC studies in human and mouse brains [156,188,189,190]. In vitro studies using primary cultured cells harvested from mice also demonstrated AHR expression in these glial cells [191,192]. Although the role of glial AHR in dioxin neurotoxicity remains poorly understood, excessive AHR signaling activation could influence neuron–glia interactions.

## 8. Conclusions and Remarks

AHR is highly conserved, not only in mammals but also among diverse animals including avian species, fish, *Drosophila*, and *Caenorhabditis elegans* [194]. In addition to rodent models, *AHR* orthologs have been reported to play crucial roles in the developmental processes of the nervous system in other vertebrate and invertebrate models. For instance, zebrafish, a model which is frequently used to evaluate the neurotoxicity of chemicals [195,196], exhibited transcriptional expression of *ahr2*, an *AHR* ortholog, in the neuroepithelium of the brain and retina during development [197]. Neurite elongation and neural circuit formation were disrupted by *ahr2* loss and CA-AHR2 expression, as well as dioxin exposure [198,199], which is consistent with the results of mouse studies [71,94,141,178,179]. Similarly, *AHR* ortholog overexpression and ablation in *Drosophila* and *C. elegans* (i.e., *spineless* and *ahr-1*, respectively) disrupted neuronal growth, including neurite elongation and the differentiation of sensory and motor neurons [200,201,202,203]. Taken together, an excess or deficiency of AHR signaling may tilt the balance of optimal neuronal growth through the dysregulation of target gene expression. Further studies on the biological roles of AHR in the nervous system across species will contribute to clarifying the molecular mechanism of AHR-dependent neurotoxicity not only in humans but also in wildlife.

In addition to chlorinated dioxins, brominated dioxins (i.e., polybrominated dibenzo-*p*-dioxins and dibenzofurans, PBDD/Fs) are produced as unwanted byproducts of the brominated flame retardants widely used in coatings and electrical applications [102,204]. They have been detected in indoor dust, industrial wastewater, food, and human samples [205,206,207,208,209,210,211,212]. Similar to PCDD/Fs, PBDD/Fs have been found to be highly persistent in the body [213], induce AHR transcriptional activation [99,100], and evoke teratogenicity [214] in rodents. Notably, experimental studies have revealed adverse effects of perinatal exposure to PBDD/Fs on behavior, although limited information is available (Table 2). Specifically, rodent offspring of pregnant dams exposed to brominated congeners exhibited impairments in learning and memory, contextual fear memory, exploratory behavior, and infant USV emissions [74,81,99,100,101]. In this context, toxicological studies on chlorinated and brominated dioxins will be important for establishing a more comprehensive estimation of environmental risks.

From a methodological perspective, transcriptomics at single-cell resolution is actively used in various fields, including toxicology [215]. Single-nucleus RNA sequencing (snRNA-seq) studies have revealed not only the toxic effects of dioxins but also the basal expression of AHR in the mouse liver [216,217]. Interestingly, central hepatocytes exhibited higher *Ahr* expression than portal hepatocytes in the lobules. This finding was in accordance with the zonal expression pattern of AHR target genes demonstrated by IHC and computational modeling [218,219]. To date, although single-cell transcriptomics for AHR expression in other organs are limited, snRNA-seq combined with spatial transcriptomics will be helpful in uncovering AHR-expressing neurons in the brain.

In conclusion, a variety of toxicological studies on AHR, demonstrating its role as a molecular intermediator between dioxins and cells, have elucidated how dioxin exposure adversely affects the functions of multiple organs and tissues, including the CNS. Accumulating evidence suggests the biological and clinical importance of AHR in regulating cellular growth and maturation, such as neurite elongation. Therefore, accurate determination of the spatio-temporal profile of AHR expression and signaling in the brain will offer valuable insights into the molecular mechanisms of dioxin neurotoxicity, advancing our understanding of the previously unreported involvement of environmental factors in brain development and function.

## Figures and Tables

**Table 1:** Toxic equivalency factors (TEFs) for dioxins and dioxin-like polychlorinated biphenyls (dl-PCBs) proposed by the World Health Organization in 1998, 2005, and 2022.

Congener	1998 TEF ^a^	2005 TEF ^b^	2022 TEF ^c^
Polychlorinated dibenzo-*p*-dioxins (PCDDs)			
2,3,7,8-TetraCDD	1	1	1
1,2,3,7,8-PentaCDD	1	1	0.4
1,2,3,4,7,8-HexaCDD	0.1	0.1	0.09
1,2,3,6,7,8-HexaCDD	0.1	0.1	0.07
1,2,3,7,8,9-HexaCDD	0.1	0.1	0.05
1,2,3,4,6,7,8-HeptaCDD	0.01	0.01	0.05
OctaCDD	0.0001	0.0003	0.001
Polychlorinated dibenzofurans (PCDFs)			
2,3,7,8-TetraCDF	0.1	0.1	0.07
1,2,3,7,8-PentaCDF	0.05	0.03	0.01
2,3,4,7,8-PentaCDF	0.5	0.3	0.1
1,2,3,4,7,8-HexaCDF	0.1	0.1	0.3
1,2,3,6,7,8-HexaCDF	0.1	0.1	0.09
1,2,3,7,8,9-HexaCDF	0.1	0.1	0.2
2,3,4,6,7,8-HexaCDF	0.1	0.1	0.1
1,2,3,4,6,7,8-HeptaCDF	0.01	0.01	0.02
1,2,3,4,7,8,9-HeptaCDF	0.01	0.01	0.1
OctaCDF	0.0001	0.0003	0.002
Non-*ortho*-substituted PCBs			
3,3′,4,4′-TetraCB (PCB 77)	0.0001	0.0001	0.0003
3,4,4′,5-TetraCB (PCB 81)	0.0001	0.0003	0.006
3,3′,4,4′,5-PentaCB (PCB 126)	0.1	0.1	0.05
3,3′,4,4′,5,5′-HexaCB (PCB 169)	0.01	0.03	0.005
Mono-*ortho*-substituted PCBs			
2,3,3′,4,4′-PentaCB (PCB 105)	0.0001	0.00003	0.00003
2,3,4,4′,5-PentaCB (PCB 114)	0.0005	0.00003	0.00003
2,3′,4,4′,5-PentaCB (PCB 118)	0.0001	0.00003	0.00003
2′,3,4,4′,5-PentaCB (PCB 123)	0.0001	0.00003	0.00003
2,3,3′,4,4′,5-HexaCB (PCB 156)	0.0005	0.00003	0.00003
2,3,3′,4,4′,5′-HexaCB (PCB 157)	0.0005	0.00003	0.00003
2,3′,4,4′,5,5′-HexaCB (PCB 167)	0.00001	0.00003	0.00003
2,3,3′,4,4′,5,5′-HeptaCB (PCB 189)	0.0001	0.00003	0.00003

^a^ Van den Berg et al., 1998 [9]. ^b^ Van den Berg et al., 2006 [11]. ^c^ DeVito et al., 2024 [10].

**Table 3:** Aryl hydrocarbon receptor expression in brain neurons of humans and rodents.

Brain Regions	Species	Methods	Markers	References
In vivo (brain tissues)				
Hippocampus				
CA1—pyramidal cell layer ^a^	Human	IHC	−	Ramos-Garcia et al., 2020 [156]
	Rat	IHC	−	Xu et al., 2016 [155]
		ISH	−	Petersen et al., 2000 [149]
	Mouse	IHC	−	de la Parra et al., 2018 [141]
		IHC	−	Bravo-Ferrer et al., 2019 [168]
		IHC	−	Song et al., 2024 [154]
		ISH	−	Kimura and Tohyama, 2017 [147]
CA3—pyramidal cell layer ^a^	Rat	IHC	−	Xu et al., 2016 [155]
		ISH	−	Petersen et al., 2000 [149]
	Mouse	IHC	−	Bravo-Ferrer et al., 2019 [168]
		IHC	−	Song et al., 2024 [154]
		ISH	−	Kimura and Tohyama, 2017 [147]
DG—granule cell layer ^a^	Rat	IHC	−	Xu et al., 2016 [155]
		IHC	−	Chen et al., 2025 [153]
		ISH	−	Petersen et al., 2000 [149]
	Mouse	IHC	−	Song et al., 2024 [154]
		FACS and RT-PCR	Nestin ^b^	Latchney et al., 2013 [97]
		ISH	−	Kimura and Tohyama, 2017 [147]
Cerebral cortex	Mouse	IHC	NeuN ^c^	Cuartero et al., 2014 [157]
Island of Calleja Major	Mouse	IHC	NeuN ^c^	Kimura et al., 2021 [148]
Locus coeruleus	Mouse	IHC	TH ^d^, DBH ^e^	Kimura et al., 2021 [148]
Ventral tegmental area	Rat	IHC	TH ^d^	Gonzalez-Barbosa et al., 2019 [159]
Substantia nigra compacta	Rat	IHC	TH ^d^	Akahoshi et al., 2009 [158]
Preoptic area	Rat	ISH	Gad1/2 ^f^	Hays et al., 2002 [152]
Rostral migratory stream	Mouse	ISH	−	Kimura and Tohyama, 2017 [147]
Embryonic brain—	Mouse	IHC	−	Abbott et al., 1995 [150]
neuroepithelium ^b^		ISH	−	Abbott et al., 1995 [150]
		ISH	−	Kimura and Tohyama, 2017 [147]
In vitro (primary cultured neurons)				
Hippocampus	Mouse	ICC and WB	−	Rzemieniec et al., 2016 [160]
		ICC and WB	MAP-2 ^g^	Rzemieniec et al., 2019 [161]
Cerebral cortex	Rat	RT-PCR and WB	−	Lin et al., 2008 [165]
	Mouse	ICC and WB	−	Kajta et al., 2009 [164]
		ICC and WB	MAP-2 ^g^	Kajta et al., 2019 [163]
Cerebellum	Mouse	ICC and WB	−	Williamson et al., 2005 [166]
		ICC	−	Dever et al., 2016 [162]

^a^ Cell layers mainly composed of neurons. ^b^ Neuronal precursors and lineage. ^c^ RNA binding protein fox-1 homolog, a neuronal marker. ^d^ Tyrosine hydroxylase, a dopaminergic and noradrenergic neuronal marker. ^e^ Dopamine-β-hydroxylase, a noradrenergic neuronal maker. ^f^ Glutamate decarboxylase 1/2, a GABAergic neuronal marker. ^g^ Microtubule associated protein 2, a neuronal marker. FACS, fluorescence-activated cell sorting; ICC, immunocytochemistry; IHC, immunohistochemistry; ISH, in situ hybridization; RT-PCR, reverse transcription PCR; WB, western blotting.

**Table 4:** AHR expression in human and rodent glial cells.

Cell Types	Species	Markers	References
In vivo (brain tissue sections stained by IHC)			
Glioma	Human	−	Opitz et al., 2011 [184]
		−	Guastella et al., 2018 [185]
		−	Takenaka et al., 2019 [187]
		−	Ma et al., 2022 [186]
Astrocyte	Human	GFAP	Rothhammer et al., 2016 [189]
		GFAP	Ramos-Garcia et al., 2020 [156]
	Mouse	GFAP	Chen et al., 2019 [188]
Microglia	Mouse	Iba1	Chen et al., 2019 [188]
		Iba1	Wang et al., 2023 [190]
In vitro (primary cultured cells stained by ICC)			
Astrocyte	Mouse	GFAP	Filbrandt et al., 2004 [193]
		GFAP	Lee et al., 2015 [191]
		GFAP	Minhas et al., 2024 [192]
Microglia	Mouse	CD11b	Lee et al., 2015 [191]

Iba1, ionized calcium-binding adapter molecule 1; ICC, immunocytochemistry; IHC, immunohistochemistry; GFAP, glial fibrillary acidic protein.

## Data Availability

Not applicable.

## Abbreviations

The following abbreviations are used in this manuscript:

AHR	Aryl hydrocarbon receptor
AHRR	Aryl hydrocarbon receptor repressor
ARNT	Aryl hydrocarbon receptor nuclear translocator
bHLH	Basic helix–loop–helix
CA-AHR	Constitutively active aryl hydrocarbon receptor
CNS	Central nervous system
CYP	Cytochrome P450
DG	Dentate gyrus
Dl-PCB	Dioxin-like polychlorinated biphenyl
GFAP	Glial fibrillary acidic protein
Iba1	Ionized calcium-binding adapter molecule 1
ICC	Immunocytochemistry
ICjM	Island of Calleja major
IHC	Immunohistochemistry
ISH	In situ hybridization
KLF6	Krüppel-like factor 6
LC	Locus coeruleus
NF-κB	Nuclear factor-κB
PAS	Per-Arnt-Sim
PBDD	Polybrominated dibenzo-*p*-dioxin
PBDF	Polybrominated dibenzofuran
PCB	Polychlorinated biphenyl
PCDD	Polychlorinated dibenzo-*p*-dioxin
PCDF	Polychlorinated dibenzofuran
RMS	Rostral migratory stream
RT-PCR	Reverse transcription PCR
snRNA-seq	Single-nucleus RNA sequencing
TeCDD	2,3,7,8-Tetrachlorodibenzo-*p*-dioxin
TEF	Toxic equivalency factor
TH	Tyrosine hydroxylase
USAF	United States Air Force
USV	Ultrasonic vocalization
XRE	Xenobiotic response element
